# Supplementary figures and images for: Preterm Birth Impedes Structural and Functional Development of Cerebellar Purkinje Cells in the Developing Baboon Cerebellum
Source: Brain Sci. 2020 Nov 24;10(12):897. doi: 10.3390/brainsci10120897 (PMC7760885; doi:10.3390/brainsci10120897)

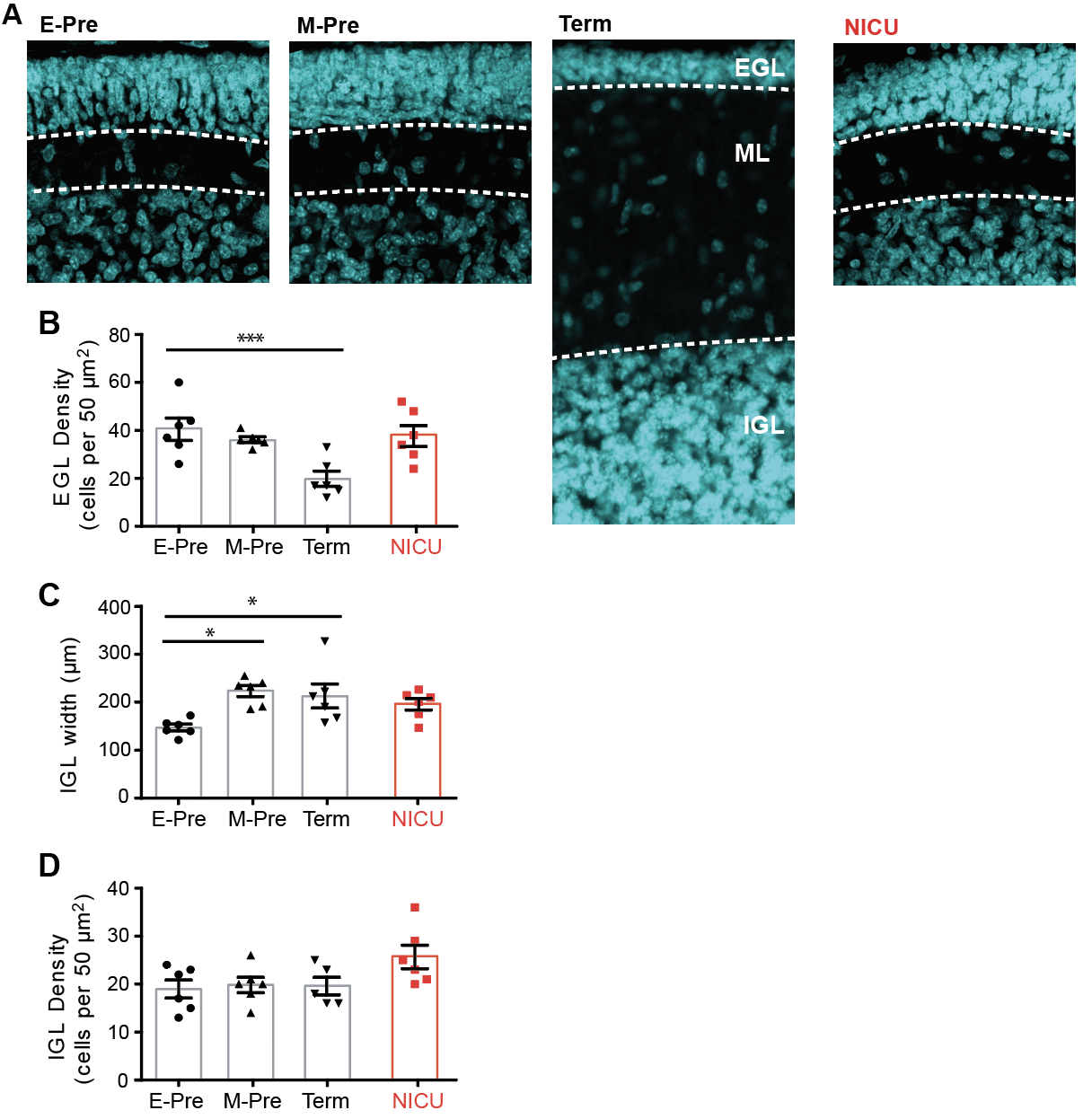

Supplement: Supplementary file 1 [file brainsci-10-00897-s001.zip › brainsci-984554-Supplementary materials.png]
